# Intervention thresholds and diagnostic thresholds in the management of osteoporosis

**DOI:** 10.1007/s40520-022-02216-7

**Published:** 2022-11-07

**Authors:** John A. Kanis, Eugene V. McCloskey, Nicholas C. Harvey, Cyrus Cooper, Rene Rizzoli, Bess Dawson-Hughes, Stefania Maggi, Jean-Yves Reginster

**Affiliations:** 1 Centre for Metabolic Bone Diseases, University of Sheffield Medical School, Beech Hill Road, Sheffield, S10 2RX UK; 2 Mary McKillop Institute for Health Research, Australian Catholic University, Melbourne, Australia; 3 Centre for Integrated Research in Musculoskeletal Ageing (CIMA), Mellanby Centre for Musculoskeletal Research, University of Sheffield, Sheffield, UK; 4 MRC Lifecourse Epidemiology Centre, University of Southampton, Southampton, SO16 6YD UK; 5 NIHR Southampton Biomedical Research Centre, University of Southampton and University Hospital Southampton NHS Foundation Trust, Tremona Road, Southampton, UK; 6 NIHR Oxford Biomedical Research Centre, University of Oxford, Oxford, UK; 7 Service of Bone Diseases, Geneva University Hospitals and Faculty of Medicine, 1211 Geneva 14, Switzerland; 8 Jean Mayer USDA Human Nutrition Research Center on Aging, Tufts University, Boston, MA USA; 9 Institute of Neuroscience, Aging Branch, CNR, Padua, Italy; 10 WHO Collaborating Center for Epidemiology of Musculoskeletal Health and Aging, Liège, Belgium; 11 Division of Public Health, Epidemiology and Health Economics, University of Liège, CHU Sart Tilman B23, 4000 Liège, Belgium

**Keywords:** Osteoporosis, Definition, Intervention thresholds, FRAX, Fracture risk, Diagnosis

Following calls to change the definition of osteoporosis [1, 2], a position paper of the International Osteoporosis Foundation (IOF) and the European Society for Clinical and Economic Aspects of Osteoporosis, Osteoarthritis and Musculoskeletal Diseases (ESCEO) recently addressed the rationale for separate diagnostic and intervention thresholds in osteoporosis [3]. The conclusions of the working group are given below.

The low rate of treatment in patients who have sustained a fragility fracture appears to underlie the calls for a change in the diagnostic criteria for osteoporosis, but there is little evidence that this alone would improve management in such patients. The WHO BMD-based, operational definition of osteoporosis is analogous to that employed successfully for the use of continuously distributed clinical risk variables in the management and prevention of other multifactorial outcomes, such as myocardial infarction (by defining hypercholesterolaemia) and stroke (by defining hypertension). It has yielded a regulatory framework in the USA, EU and elsewhere which has permitted the development of an enviable armamentarium of therapeutic interventions.

The confusion appears to arise because of the erroneous conflation of diagnostic and intervention thresholds. The example used for the basis of the paper by Paskins and colleagues [2] illustrates this clearly, namely a 76-year-old woman with a recent vertebral fracture. Here, the diagnosis is one of fragility fracture, which like a diagnosis of myocardial infarction or stroke, should initiate a course of interventions, including pharmacological agents, to reduce future risk of recurrence. The need for a parallel diagnosis of BMD-defined osteoporosis serves to delay and indeed limit access to treatment, particularly where the result is misinterpreted, possibly fuelled by the previous misconceptions that the treatments do not work in the absence of BMD-defined osteoporosis [4, 5]. Importantly, there is increasing evidence that the implementation of fracture liaison services though campaigns, such as Capture the Fracture can improve access to better management and treatment leading to reductions in future fractures [6].

It is widely recognised that BMD alone for fracture risk assessment is less sensitive than risk assessment algorithms, such as FRAX® that incorporate risk indicators in addition to BMD [7]. It is certainly relevant to question the need for diagnostic criteria when the field is moving towards risk-based assessment and intervention, including adjustments to FRAX and guidance thresholds to distinguish high risk from very high risk to optimise the use of anabolic agents [8–12]. These developments will inevitably decrease the clinical utility of the T-score, but they will, however, take time to implement into routine clinical practice. Notwithstanding, the current diagnostic criteria will remain of value in quantifying the burden of disease and the development of strategies to combat osteoporosis in the foreseeable future.

It is hard to argue that operational BMD-based definition is anything other than a triumph in healthcare, and there appears little possible (or indeed intellectually sound) reason to argue for a change. Those suggesting an alteration to the diagnostic criteria for osteoporosis would do well to consider the implications of such an approach if it were to be adopted more widely. Would they really be happy with diagnosing hypertension purely on the basis of a stroke or myocardial infarction? In our view, the proposal is intellectually constrained, inadequately justified and may well inappropriately reflect the pressures of reimbursement led healthcare.

We recommend that the BMD-based definition of osteoporosis be retained whilst further clarity is brought to bear on the distinction between BMD-based diagnoses and intervention thresholds.
